# Mini-open versus percutaneous surgical repair for acute Achilles tendon rupture: a systematic review and meta-analysis

**DOI:** 10.1007/s00264-024-06362-7

**Published:** 2024-10-28

**Authors:** Marian Andrei Melinte, Dan Viorel Nistor, Rodrigo Arruda de Souza Conde, Ricardo González Hernández, Prajna Wijaya, Kabuye Marvin, Alexia Nicola Moldovan, Razvan Marian Melinte

**Affiliations:** 1 Pharmacy, Science, and Technology of Targu Mures, “George Emil Palade” University of Medicine, Strada Rasaritului nr. 10, Targu Mures, 540143 Romania; 2 1st Department of Orthopedics and Traumatology, “Iuliu Hațieganu” University of Medicine and Pharmacy, 8 Victor Babes Street, Cluj-Napoca, 400012 Romania; 3 Fundación Barceló - Instituto Universitario de Ciencias de la Salud, Buenos Aires, Argentina; 4 Department of Orthopedics and Traumatology, Clinica General Del Norte, 70th Street 35th Avenue, Barranquilla, 080020 Colombia; 5 Faculty of Medicine, Universitas Indonesia, Jakarta, 40115 Indonesia; 6 Faculty of Medicine, Azerbaijan Medical University, Samad Vurghun, Baku, Nasimi, AZ1022 Azerbaijan; 7 Department of Orthopedics, Regina Maria Health Network, 49 Gheorghe Marinescu Street, Targu Mures, 540136 Romania; 8 MedLife Humanitas Hospital, 75 Frunzisului Street, Cluj-Napoca, 400664 Romania

**Keywords:** Achilles, Mini-open, Limited open, Percutaneous, Meta-analysis, AOFAS

## Abstract

**Purpose:**

To compare the clinical outcomes and complications of mini-open (MOT) and percutaneous techniques (PT) in the surgical repair of acute Achilles tendon rupture (AATR).

**Methods:**

We systematically searched PubMed, Scopus, Web of Science, Clinical Trials, and the Cochrane Library for studies comparing MOT with PT for AATR. We assessed functional outcomes, complication rates, and operation time. Statistical analyses were performed using RevMan Web. Odds ratios (ORs) and mean difference (MD) with 95% confidence intervals (CIs) were pooled with a fixed-effects model for dichotomous and continuous endpoints, respectively. Heterogeneity was evaluated with I^2^ statistics.

**Results:**

Eight studies, comprising 484 patients, were included, of whom 226 (46%) underwent MOT. MOT was associated with a significantly lower re-rupture rate (1.48% vs. 6.11%; OR 0.28; 95% CI 0.09–0.86; *p* = 0.03; I^2^ = 6%) and sural nerve injury rate (0.57% vs. 5.64%; OR 0.24; 95% CI 0.07–0.81; *p* = 0.02; I^2^ = 0%). No significant differences were observed in venous thrombosis (OR 0.81; 95% CI 0.17–3.94; *p* = 0.33; I^2^ = 0%), wound infection (OR 0.56; 95% CI 0.12–2.62; *p* = 0.46; I^2^ = 0%), or operation time (MD 1.83 min; 95% CI -1.13–4.79; *p* = 0.23; I^2^ = 88%). Functional outcomes showed higher American Orthopaedic Foot and Ankle Society (AOFAS) Ankle-Hindfoot scores in the MOT group (MD 1.52 points; 95% CI 0.62–2.42; *p* = 0.001; I^2^ = 3%), while (Achilles Tendon Total Rupture Score) ATRS, time to return to activities, and ankle plantar and dorsiflexion were comparable.

**Conclusions:**

MOT for AATR repair seems to reduce re-ruptures and sural nerve injuries while improving AOFAS scores, with no significant differences in other complications compared to PT. These findings suggest that the MOT may offer a safer and equally effective alternative to PT for AATR.

**Supplementary Information:**

The online version contains supplementary material available at 10.1007/s00264-024-06362-7.

## Introduction

The Achilles is the strongest tendon in the human body and transfers the force from the gastrocnemius and soleus muscles to the calcaneus [1]. Acute Achilles tendon rupture (AATR) is one of the most frequent musculoskeletal injuries, with an annual incidence of 2.5 to 47 events per 100,000 persons, an increasing rate in European populations in the last 20 years, and a high degree of variability [2–6]. As such, it is a source of potentially severe disability. This injury results in significant functional impairment, pain, and disability, necessitating effective treatment to restore optimal functionality and prevent long-term complications [7].

Management of AATR has traditionally consisted of three main treatment options: nonsurgical, classic open surgery, and minimally invasive techniques (MIT). Surgical repair has shown favor compared to conservative treatment, having lower re-rupture rates and quicker return to sports, making it the preferred choice, particularly for those with high physical demands [5, 8–10]. Classic open surgical repair, while effective, has been associated with complications such as wound infections and necrosis, resulting in devastating soft tissue complications. To reduce incision complications, the first type of MIT, the percutaneous technique (PT), was introduced in 1977 by Ma and Griffith [11]. Since then, many mini-invasive techniques have been developed such as the mini-open technique (MOT), AchillonSystem™ and Percutaneous Achilles Repair System (PARS) by Arthrex^Ⓡ^.

Previous meta-analyses have shown that percutaneous surgery has better outcomes than traditional open techniques, including shorter surgery duration, a lower rate of post-operative wound necrosis, superficial and deep tissue infections, and scar tissue adhesions [12, 13]. However, a risk of sural nerve injury of up to 14% has been reported [14]. Intraoperative ultrasound-guided approaches can be used with PT to reduce this risk and have shown potential benefits [15], but the literature suggests that it can’t completely mitigate it [16, 17]. MOT has been developed to combine the advantages of percutaneous and open repair. It allows direct visualization of the Achilles tendon repair and has been hypothesized to reduce the risk of trapping the sural nerve. Ultrasound guidance can also be used here to identify the ruptured tendon ends.

Nevertheless, the literature remains inconclusive regarding the superiority of MOT over PT as the minimally invasive treatment of choice for AATR. While some studies suggest better functional outcomes and lower complication rates with MOT, others report comparable results between the two techniques as PT repair is improved to avoid sural nerve injury [14, 18, 19]. To address this controversy, we performed a systematic review and meta-analysis to evaluate the efficacy and safety of MOT and PT for AATR.

## Methods

This systematic review and meta-analysis followed the Cochrane Collaboration Handbook [20] for Systematic Reviews of Interventions and the Preferred Reporting Items for Systematic Reviews and Meta-Analysis (PRISMA) [21] Statement guidelines. This meta-analysis was prospectively registered in the International Prospective Register of Systematic Reviews (PROSPERO) under the unique identifier CRD42024538923.

### Eligibility criteria

Inclusion in this meta-analysis was restricted to studies that met all the following eligibility criteria: (1) studies including patients with isolated AATR, (2) comparing the MOT with PT, and (3) reporting at least one outcome of interest. We excluded studies (1) with inadequate or no control group, (2) involving non-human, cadaveric, or in-vitro subjects, (3) including patients with chronic Achilles rupture, or (4) did not have a full-text report.

### Definitions

For the MOT group, the term used in this paper does not necessarily reflect the terms in the studies included, as authors have used terms like “minimally invasive repair” or “modified mini-invasive repair.*”* As there is no definitive consensus, we included studies that had the following surgical features: (1) incision at the level of tendon rupture and (2) direct visualization of the repair or the Achilles tendon.

PT is defined as the technique first described by Ma and Griffith [11]. As noted in previous studies [18, 22, 23], this category includes variations and different suture techniques such as Bunnell, Krakow, or devices like Tenolig. The term PT used in this paper is consistent with the terminology used in the included studies.

### Search strategy

We systematically searched PubMed, Scopus, Web of Science, Clinical Trials, and the Cochrane Library from inception to May 2024. After removing duplicates, two authors (M.A. M. and K.M.) screened titles and abstracts and independently assessed full-text articles for inclusion based on prespecified criteria. Discrepancies were resolved through consensus, including a third author (P.W.). Additionally, we reviewed references from included articles and previous systematic reviews to identify relevant studies. Moreover, we used an artificial intelligence powered tool, Research Rabbitt, to find related articles.

Our search strategy included the following medical subject heading terms: “Achilles,” “Achilles Tendon“[Mesh], “AATR,” “heel cord,” “calcaneus tendon,” “mini-open,” “limited-open,” “MOA,” “minimal incision,” “mini-incision,” “internal splinting,” “Achillon,” “small incision surgery,” “limited access surgery,” “mini-invasive,” “minimally-invasive,” “minimum invasive,” “percutaneous,” “Griffith,” “Griffiths,” “Webb,” “keyhole surgery,” “image-guided surgery.” The exact terms applied in each database from this search strategy are shown in Supplementary Appendix.

### Endpoints

Functional outcomes included (1) American Orthopaedic Foot and Ankle Society (AOFAS) score; (2) Achilles tendon Total Rupture Score (ATRS); (3) time to return to previous activities; (4) ankle plantar and dorsi flexion. Outcomes that reflected complication rates were (1) sural nerve injury, (2) re-rupture of the Achilles tendon, (3) venous thrombosis, and (4) wound infection. Operation time was also collected due to availability in the included studies.

### Statistical analysis

Odds ratios (ORs) with 95% confidence intervals (CIs) were calculated to compare the incidence of binary endpoints. Continuous outcomes were compared using the mean difference (MD) with 95% CIs. Heterogeneity was assessed using Cochran’s Q statistic and Higgins & Thompson’s I² statistic, quantifying the proportion of total variation across studies due to heterogeneity. We interpreted heterogeneity as low if *P* > 0.10 and I² < 25%. The choice between fixed-effects and random-effects models was driven primarily by heterogeneity. A fixed-effects model was used when heterogeneity was deemed low (*P* > 0.10 and I² < 25%). Conversely, the DerSimonian-Laird random-effects model was used for outcomes with moderate to high heterogeneity (I² ≥ 25%), accounting for between-study variability. Statistical significance was defined as *P* < 0.05, indicating that results with P values below this threshold represent a statistically significant synthesis of outcomes across the included studies. All statistical analyses were conducted using Review Manager Web (Nordic Cochrane Centre, The Cochrane Collaboration, Copenhagen, Denmark). Additionally, for outcomes with very few events (< 1%), a Peto fixed-effects model was applied, as recommended by Cochrane, to provide more accurate estimates when event rates are extremely low [20].

### Quality assessment and publication bias

Observational studies were appraised using the Risk of Bias Summary for Non-randomized Studies (ROBINS-I) to assess the methodological quality of included studies, a tool based on answers to the signaling questions, judgments for each bias domain, and for overall risk of bias, which allows labeling each study as “low,” “moderate,” “serious,” or “critical” risk of bias. Two independent investigators (M.A.M. and P.W.) performed the assessment, and disagreements were resolved by consensus. Funnel plots of individual study weights against point estimates were used to check for evidence of publication bias. As per Cochrane’s recommendations, Egger’s test was not performed due to the number of included studies in this meta-analysis (*n* < 10) [20].

## Results

### Study selection and characteristics

**Fig. 1:**
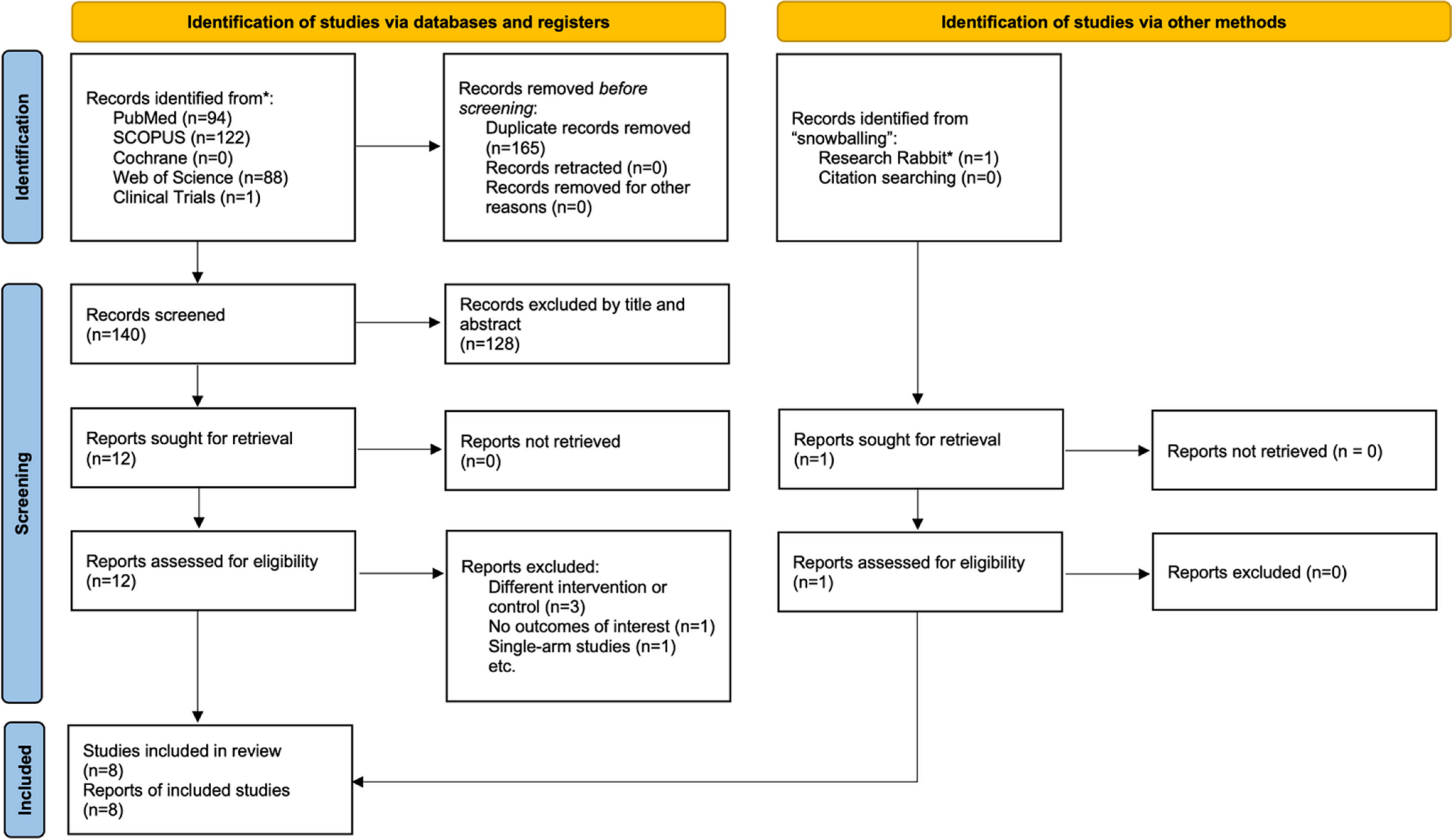
PRISMA 2020 flow diagram for new systematic reviews which included searches of databases, and other sources. Research Rabbit* is an artificial intelligence (AI) based tool that was used to do “backward snowballing” on all included papers. From: Page MJ, McKenzie JE, Bossuyt PM, Boutron I, Hoffmann TC, Mulrow CD, et al. The PRISMA 2020 statement: an updated guideline for reporting systematic reviews. BMJ 2021;372:n71. 10.1136/bmj.n71

As detailed in Fig. 1, 295 studies were identified using the search strategy in the five databases and manual search of references of pertinent reviews and meta-analyses. After removing duplicate articles and unrelated studies, 12 were thoroughly reviewed for the inclusion and exclusion criteria. Eight observational studies and 484 patients were included, of whom 226 (46%) underwent MOT. Population characteristics are presented in Table 1. The studies were heterogeneous regarding the surgical technique, rehabilitation protocol, follow-up period, and timing of intervention. (Tables 1 and 2)

**Table 1:** Baseline characteristics of included studies

Study	Design	Patients MOT/PT	Male (%) MOT/PT	Age^†^ (years) MOT/PT	BMI^†^ MOT/PT	Days to surgery_†_ MOT/PT	No exercise habits (%)	Follow-up^†^ (months)
Laboute 2023	Prospective Multicenter Cohort	15/22	73/90	43.2/43.7	25.9^*^	NA	19	6^‡^
Liu 2020	Prospective Cohort	70/44	87/ 82	40.2/38.2	23.6/ 23.9	4.6/4.5	NA	3, 6, 12, 24^‡^
Cecarelli 2007	Retrospective Power Analysis	12/12	100/75	40.4/44.7	N/A	7^§*^	NA	33 (24–42)
Jiang 2021	Retrospective Cohort	24/29	96/90	32.2/33.3	N/A	5.4/5.3	13	60 (48–67)
Inci 2022	Retrospective Cohort	22/20	91/90	39.5/41.2	N/A	3.0/3.2	12	31.5 (20–42)
Li 2021	Retrospective Cohort	34/34	91/91	32.3/30.5	24.3/ 23.5	14^§*^	NA	28.4
Magnitskaya 2024	Retrospective Cohort	19/74	NA	40^*^	N/A	NA	NA	84 (60–120)
Subaşı 2023	Retrospective Cohort	30/23	63/ 65	45.2/44.2	24.4/ 24.9	NA	NA	9^‡^

Abbreviations: BMI: Body mass index; IQR: Inter-quartile range; MOT: Mini-open technique; NA: not available; PT: Percutaneous Technique

§ maximum; * For both groups; † mean or median (IQR); ‡ Absolute value

**Table 2:** Study intervention types and the rehabilitation protocol

Study	Mini-open		Percutaneous		Post-operative protocol*
	Technique	Suture	Technique	Suture	
Cecarelli 2007	Prone, tourniquet. Longitudinal medial paratendinous incision (2–3 cm). Paratenon incised, proximal stump identified, Achillon^®^ device used.	Three Number 1 absorbable sutures via Achillon^®^. Sutures knotted in equinus position. Paratenon and skin closed with 3 − 0 resorbable sutures.	Prone, knee flexed, tourniquet optional. 5-mm incisions at lesion, distal, and proximal sites. Paratenon opened with small forceps.	Modified Ma and Griffith. Two Number 2 Bunnell-type bioresorbable sutures. Sutures knotted in equinus position. 3 − 0 resorbable sutures for skin.	Boot maintained for 8 weeks 0–2 weeks: Rest, no weightbearing. 3–4 weeks: Partial load (up to 15 kg), home cycling. 4–6 weeks: Boot in neutral, active motion (0° to 30° plantar flexion). 6–8 weeks: Partial to full load (up to 25 kg), active motion (5° dorsiflexion). 8 weeks+: Full load, intensive ankle exercises.
Jiang 2021	Prone, tourniquet. 2-cm incision, paratenon incised, calcaneal 5-mm incision, 3-cm incision. Suture anchor used.	Krackow technique, absorbable sutures, knots embedded. Paratenon and incision closed.	Prone. 2–3 cm incision, paratenon incised, Allis clamps, bent oval forceps.	High-strength sutures, Keith needle, Krackow stitch, absorbable sutures. Paratenon and incision closed.	0–2 weeks: short brace at 20° equinus, knee exercises. 2 weeks+: Full weightbearing with boot. 3 months: Jogging, low-impact activities. 6 months: Full preinjury activity.
Inci 2022	Prone, Transverse/longitudinal 2–3 cm incision, paratenon opened, tendon ends clamped.	Ethibond Excel 2.0, sutures tied in plantar flexion. Paratenon and incision closed.	Prone. Two step incisions, paratenon retracted.	Ethibond Excel 2.0, suture hook, sutures tied in plantar flexion. Paratenon and incision closed.	0–2 weeks: Boot at 30° plantar flexion, no weightbearing, knee exercises. 2–6 weeks: Reduce plantar flexion by 10° every 2 weeks, partial weightbearing. 3 weeks+: Active dorsiflexion. 6 weeks+: Remove boot, full weightbearing, active resistance and stretching exercises.
Laboute 2023	Small incision over tendon, visual control, specific devices (Achillon^®^, Akilink^®^, PARS^®^) or none.	Achillon^®^, Akilink^®^, PARS^®^, modified Maffulli, Ma & Griffith.	Tenolig^®^ (TL) technique. Two incisions, harpoon-loaded needles, externalized, skin buttons.	TL technique, harpoons, fixed with skin buttons.	0–3 weeks: Ankle immobilized in equinus, non-weightbearing. 3–6 weeks: Partial weightbearing with crutches, equinus gradually lowered to 0°. 6–9 weeks: Full weightbearing with 15 mm heel-piece. 9 weeks+: Cardiovascular training, muscle strengthening. 4–6 months: Plyometric exercises, jogging.
Liu 2020	Prone, 2–3 cm incision, paratenon incised, tendon clamped, direct visualization.	Ethibond No. 2, figure-of-eight, knots buried. Paratenon and tissue closed.	Prone. 2-cm incision, Achillon^®^ device, sutures passed through paratenon.	Achillon^®^ technique, three sutures, tightened, subcutaneous tissues and skin closed.	0–2 weeks: Plantar flexed short leg cast, partial weightbearing. 3–6 weeks: Adjustable orthosis, 10–30° plantar flexion, full weightbearing with crutches. 6 weeks+: Walking without orthosis, full range of motion exercises. 16 weeks: Running and sports training.
Li 2021	Prone, epidural anesthesia. 2–3 cm transverse incision, tendon exposed, proximal end clamped, blunt separation with channel instrument.	Proximal/distal sutures with guide needle, tension knotted, absorbable Vicryl Suture 3 − 0. Incision sutured, leg fixed in plaster.	Prone, tourniquet. Ma-Griffith technique, adaptations to minimize sural nerve damage.	Ma-Griffith technique, specific details not provided.	0–2 weeks: Non-weightbearing equinus cast (20°-25°). 3–10 weeks: Walker boot, range-of-motion exercises, muscle strengthening, gait restoration. 10 weeks+: Ankle flexibility and muscle strength improvement, full ankle movement. 24 months: Follow-up, functional evaluation with AOFAS and ATRS scores.
Magnitskaya 2024	Transverse approach (up to 4 cm), assess tendon ends and knots.	NA	Bunnel-Cuneo suture, tying and immersion knots, two punctures.	Bunnel-Cuneo suture.	0–3 weeks: Immobilization in equinus position. 4–6 weeks: Immobilization in neutral position, limited axial load.
Subaşı 2023	Prone. 2-cm transverse incision, paratenon developed, tendon ends observed.	Bunnell technique, sutures tied in plantar flexion. Paratenon closed.	Prone. Two stab incisions, suture passed with straight needle.	Bunnell technique, sutures tied near tendon.	0–2 weeks: non-weightbearing, short-leg cast at 20° plantar flexion, knee flexion and extension exercises. 3 weeks: Active dorsiflexion. 6 weeks+: Active resistance and stretching exercises, full weightbearing. 1 month: ATRS and AOFAS scores evaluated. 6 months: ATRS, AOFAS, and TAS scores evaluated, return to daily activities.

*For both groups; NA – Not available; TL – Tenolig^®^

### Complication rates

**Fig. 2:**
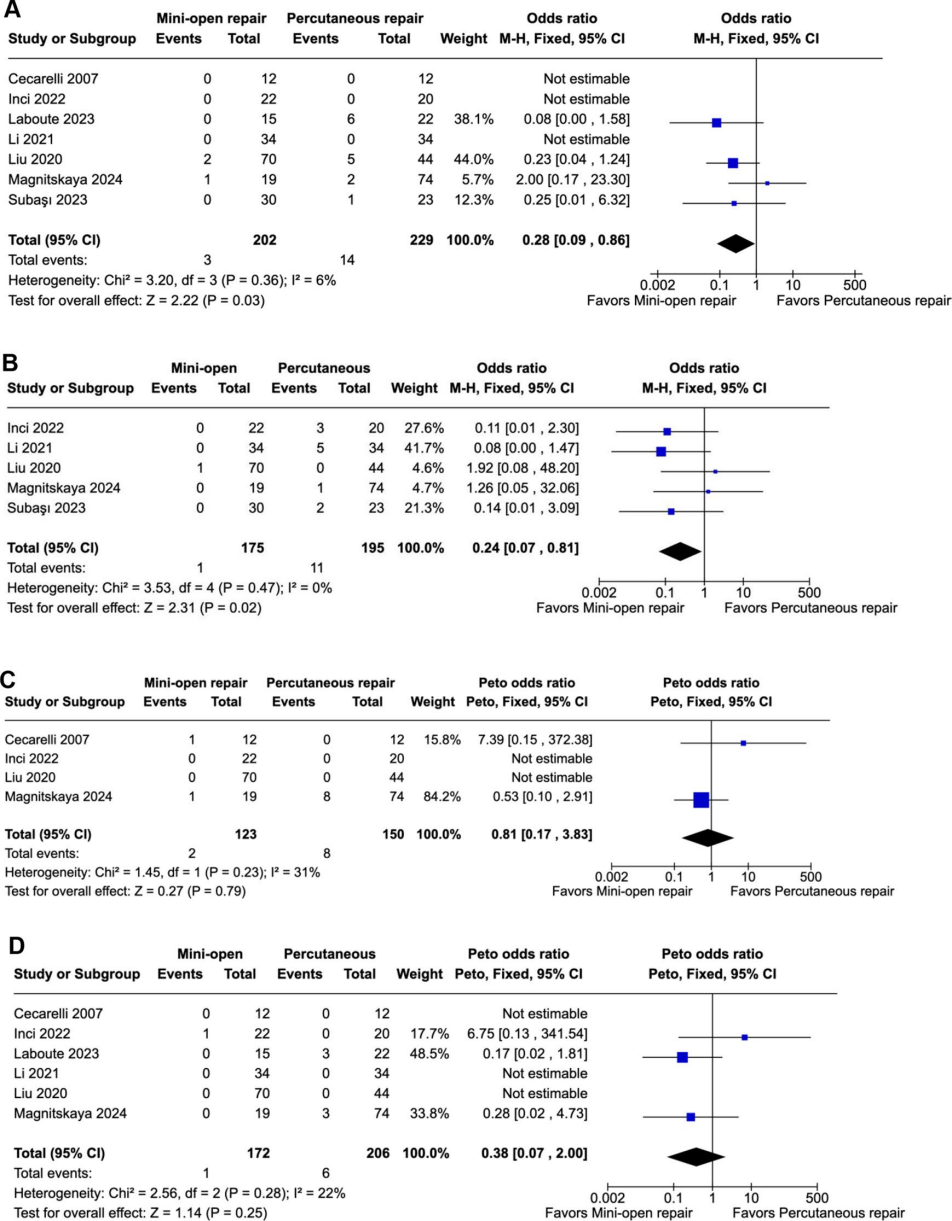
**A** In patients with acute Achilles tendon rupture, the mini-open technique was associated with reduced re-ruptures compared to the percutaneous technique. **B** In patients with acute Achilles tendon rupture, the mini-open technique was associated with reduced sural nerve injuries compared to the percutaneous technique. **C** Peto fixed-effect model showed that in patients with acute Achilles tendon rupture, there was no significant difference in venous thrombosis between the mini-open and the percutaneous technique. **D** Peto fixed-effect model showed that in patients with acute Achilles tendon rupture, there was no significant difference in wound infection (superficial and deep) between the mini-open and the percutaneous technique

MOT significantly reduced re-rupture rates (1.48%) compared with the PT group (6.11%) (OR 0.28; 95% CI 0.09–0.86; *p* = 0.03; I²=6%; Fig. 2A). Moreover, the sural nerve injury rate was also significantly lower in the MOT group (0.57%) compared with the PT group (5.64%) (OR 0.24; 95% CI 0.07–0.81; *p* = 0.02; I²=0%; Fig. 2B). There was no difference regarding venous thrombosis (OR 0.81; 95% CI 0.17–3.83; *p* = 0.79; I²=31%; Fig. 2C) or wound infection (OR 0.38; 95% CI 0.07–2.00; *p* = 0.25; I²=22%; Fig. 2D).

### Functional outcomes and operating time

**Fig. 3:**
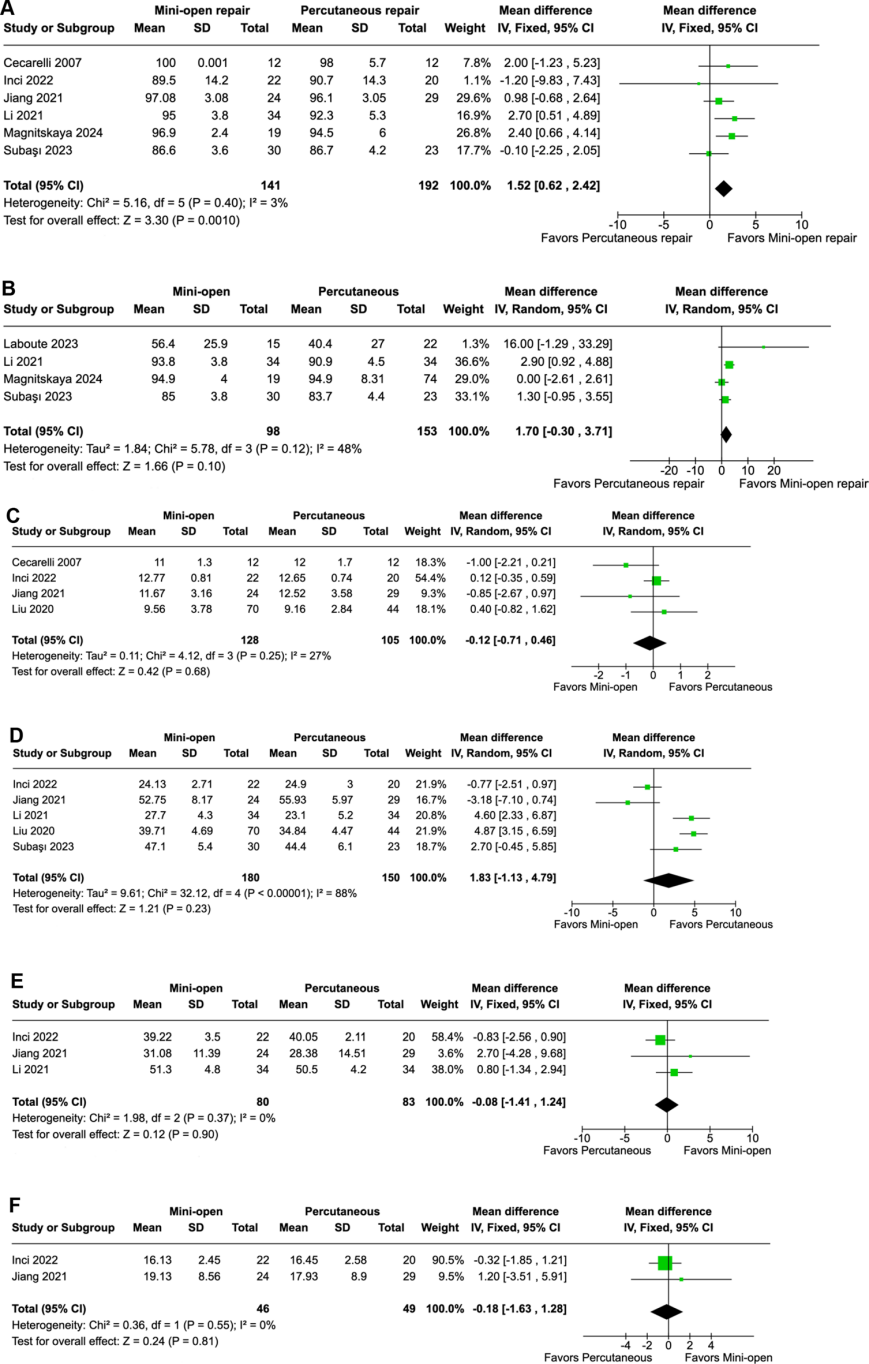
**A** In patients with acute Achilles tendon rupture, the AOFAS score was significantly higher in the mini-open group compared to the percutaneous group. **B** In patients with acute Achilles tendon rupture, the ATRS score was not different between the mini-open group and the percutaneous group. **C** In patients with acute Achilles tendon rupture, the time to return to previous activity was similar between the mini-open group and the percutaneous group. **D** In patients with acute Achilles tendon rupture, operating time was similar between the mini-open group and the percutaneous group. **E** In patients with acute Achilles tendon rupture, ankle plantar flexion was similar between the mini-open group and the percutaneous group. **F** In patients with acute Achilles tendon rupture, ankle dorsiflexion was similar between the mini-open group and the percutaneous group

The MOT group had significantly higher AOFAS scores than the PT group (MD 1.52 points; 95% CI 0.62–2.42; *p* = 0.001; I²=3%; Fig. 3A). However, there was no difference in ATRS score (MD 1.70 points; 95% CI -0.30–3.71; *p* = 0.10; I²=48%; Fig. 3B), time to return to previous activities (MD -0.12 weeks; 95% CI -0.71–0.46; *p* = 0.68; I²=27%; Fig. 3C), ankle plantar flexion (MD -0.08 degrees; 95% CI -1.41–1.24; *p* = 0.90; I²=0%; Fig. 3E), and ankle dorsiflexion (MD -0.18 degrees; 95% CI -1.63–1.28; *p* = 0.81; I²=0%; Fig. 3F). We found no difference in operating time between groups (MD 1.83 min; 95% CI -1.13–4.79; *p* = 0.23; I²=88%; Fig. 3D).

### Quality assessment and publication bias

Three non-randomized studies were considered to have a serious risk of bias in the domains of “confounding,” “selection of participants,” and “selection of the reported result.” Other studies were also considered to have a moderate risk of bias in the “selection of the reported” result domain due to the subjective nature of the AOFAS score. Individual appraisal of each study with the ROBINS-I tool was made and found in Table 3. Although limited by the small number of studies, there was no definitive evidence of publication bias in the funnel plots. (Supplementary Appendix)

**Table 3:**
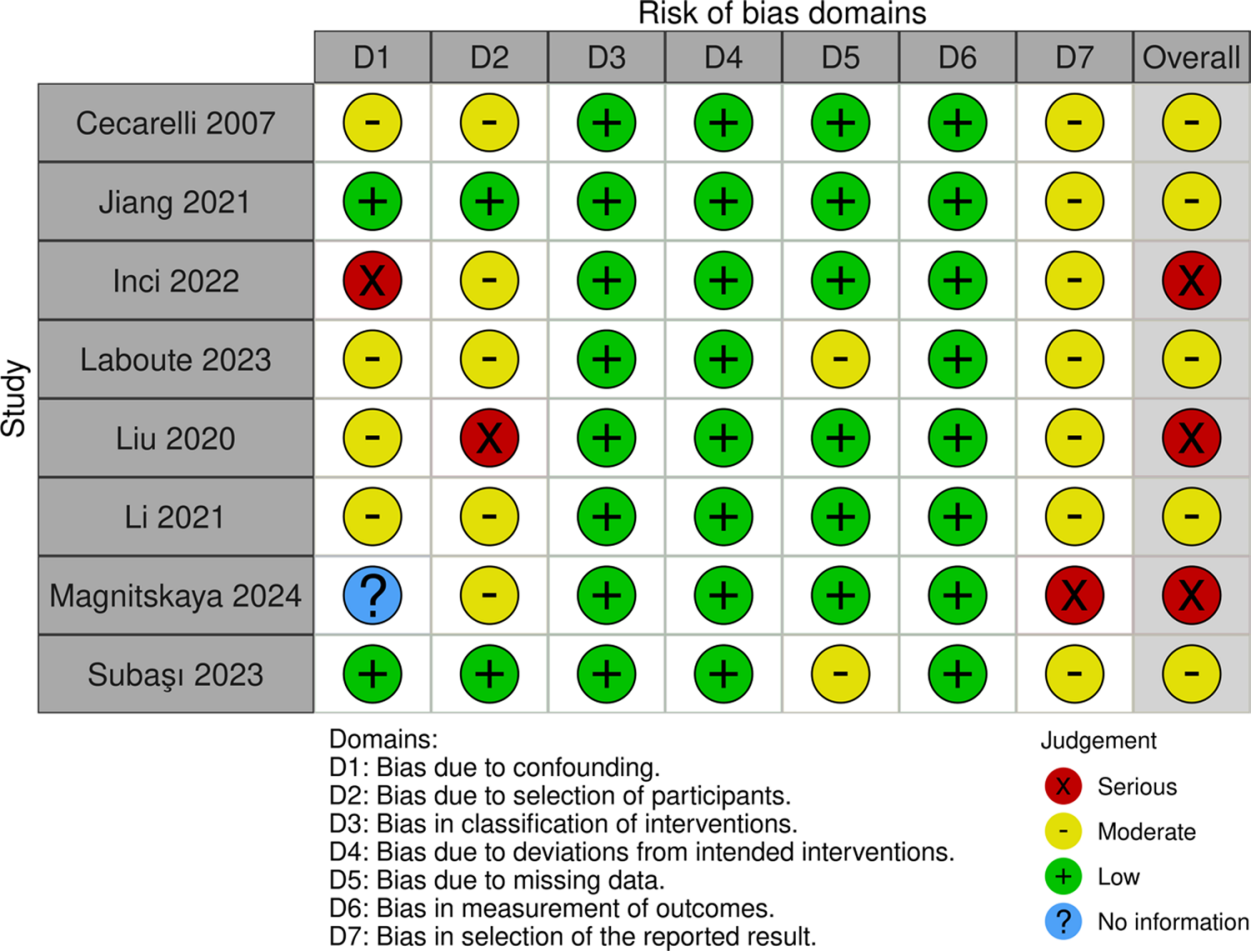
Critical appraisal of individual studies according to the Cochrane Collaboration’s ROBINS- I tool for assessing risk of bias in non-randomized trials

## Discussion

In this systematic review and meta-analysis of eight studies and 484 patients, we compared the MOT with PT for the mini-invasive repair of AATR. Our main findings include: (1) re-ruptures were significantly lower in the MOT group (1,48% vs. 6,11%); (2) MOT was associated with a reduction in sural nerve injury rates (0,54% vs. 5,67%); (3) wound infections were rare with no significant difference; (4) venous thrombosis were exceedingly rare except for one study [24]; (5) MOT was associated with a slightly higher AOFAS score (1,52 points); (6) other functional outcomes such as ATRS, time to return to previous activities, and ankle plantar and dorsiflexion angles were improved but comparable between the techniques. To the best of our knowledge, this meta-analysis is the first to compare MOT with PT in AATR patients, as previous work has focused on different mini-invasive procedures versus classical open repair [12, 25, 26] or compared multiple treatment strategies [10].

Surgical versus nonsurgical repair of acute Achilles tendon ruptures has been extensively studied. While non-operative treatments avoid surgical risks, they often result in higher re-rupture rates and require longer rehabilitation periods. In contrast, operative repair, particularly using minimally invasive techniques, typically shows lower re-rupture rates and faster recovery times. Previous studies have suggested that surgical intervention is preferred for active patients due to better functional outcomes and reduced re-rupture rates [5, 10].

PT were developed to minimize complications associated with open surgery, such as wound infections and soft tissue damage, and to shorten surgery duration and reduce anaesthesia risks [11]. However, they involve small incisions and blind suturing, which initially led to higher risks of sural nerve injury and re-rupture due to non-anatomical tendon contact [27]. Modifications, including ultrasound guidance, specific suture patterns, and lateral incisions, have improved repair strength and reduced complications [1, 14, 18, 28]. The MOT combines the benefits of percutaneous and open repairs by using a small longitudinal incision for direct visualization and accurate suturing, ensuring proper tendon alignment [1, 27, 29, 30]. Variations like the limited-open technique offer enhanced tendon visualization with slightly larger incisions [1, 14], while internal splinting with FiberTape provides additional support during healing [1, 23]. Hybrid devices like Achillon^®^ and PARS by Arthrex, used in both mini-open and percutaneous techniques, also contribute to improved outcomes [18, 19, 31].

The re-rupture rate for PT in the literature has been reported to be between 2.6 and 16.7% [12, 32, 33]. In our meta-analysis, all studies were in that range except for one study with an incidence of 27% with the Tenoligdevice [22]. Even though earlier studies initially did not find re-ruptures with the Tenolig [34], later publications had similar results at around 20% [35]. While the literature suggests that results seemed related to the surgeons’ expertise, according to Soubeyrand’s research, 45% of needles were incorrectly positioned in percutaneous Achilles tendon repairs [22, 23, 36]. So, this causes the sutures to be in incorrect placement, possibly creating a re-rupture. The contrast between the Tenolig and the other techniques may also be explained by the reinforced sutures (Bunnel-Cuneo, Krackow). MOT, as it allows for direct visualization, can ensure that no residual gap between the two tendon stumps is present at the moment of repair.

PT carries a higher risk of sural nerve entrapment, even with minimally invasive instruments or intra-operative ultrasonography [16, 17]. Initially, under the Ma–Griffith percutaneous technique, a sural nerve palsy rate reaching 60% has been reported [14]. Surgical advancements have decreased the incidence to between 0% and 27% [23, 33]. This meta-analysis included studies that, in most cases, accounted for technique variations that would minimize this risk, such as close-together incisions or modified Ma-Griffith technique, as described in Table 2. The pooled result from our meta-analysis showed an incidence of 5.6%, aligning as expected with the lower end of reported data. In contrast, the MOT group showed a significantly lower incidence at 0.57%.

Cecarelli et al. treated 12 patients using the MOT, achieving a follow-up AOFAS score of 100 points [18]. Similarly, Jiang et al. performed PT repair on 96 patients, reporting an AOFAS score of 96.1 [23]. Our meta-analysis showed high AOFAS scores for both groups but significantly higher for the MOT group, with a mean difference of 1.52 points. This can be explained by the fact that as the mini-open repair system reduces the number of suture knots, it lowers the sensation of foreign bodies from the suture knots and keloid formation and consequently can improve both the function and appearance of the hindfoot [14]. Our results suggest that MOT and PT can achieve satisfactory functional outcomes in patients with AATR, with a slight advantage observed for the MOT.

This study has several limitations. First, the small sample size and reduced power in included studies may limit our finding’s generalizability. Second, the heterogeneity in surgical techniques, rehabilitation protocols, and follow-up durations across studies introduces confounding that could impact the results. Differences in the rehabilitation regimens, such as early range of motion, timing of weightbearing, or distinguishing between treatment arms, could impact functional outcomes and complication rates. Third, scarce outcome availability might make this study underpowered to detect small but significant changes in ATRS score and ankle mobility differences between MOT and PT. Fourth, while statistically significant, the minimal difference in AOFAS scores may not be clinically meaningful. Finally, since this meta-analysis is based only on observational data, more randomized controlled trials are needed. Larger studies with subgroup analyses, primarily focusing on elderly patients and athletes with different recovery rates, are necessary to confirm our results. Despite these limitations, we employed a robust methodological approach and strict eligibility criteria, including the Peto fixed-effects model for rare events, to enhance the reliability of our findings.

## Conclusion

This meta-analysis indicates that the MOT for AATR repair may result in lower re-rupture and sural nerve injury rates compared with PT. Both techniques can achieve satisfactory functional outcomes. However, the MOT was associated with a slightly higher AOFAS score, suggesting a potential advantage in functional recovery.

## Electronic supplementary material

Below is the link to the electronic supplementary material.

Supplementary Material 1

## Data Availability

The raw data are available for future needs and can be obtained by contacting the corresponding author.

## Abbreviations

AATR: Acute Achilles Tendon Rupture
AOFAS: American Orthopaedic Foot and Ankle Society
ATRS: The Achilles Tendon Total Rupture Score
MD: Mean Difference
MIT: Mini–Invasive Technique/s
MOT: Mini–Open Technique
OR: Odds Ratio
PARS: Percutaneous Achilles Repair System
PRISMA: Preferred Reporting Items for Systematic Reviews and Meta–Analysis
PROSPERO: Prospective Register of Systematic Reviews
PT: Percutaneous Technique
TL: Tenolig^®^ System
