# Supplementary material for: Mini-open versus percutaneous surgical repair for acute Achilles tendon rupture: a systematic review and meta-analysis
Source: Int Orthop. 2024 Oct 28;49(1):259–69. doi: 10.1007/s00264-024-06362-7 (PMC11703899; doi:10.1007/s00264-024-06362-7)

Supplementary Appendix

**Search strategy:**

**PubMed (MEDLINE):**

(Achilles OR "Achilles Tendon"[Mesh] OR AATR OR “heel cord” OR “calcaneus tendon”) AND (“mini-open” OR “limited-open” OR MOA OR “minimal incision” OR “mini-incision” OR “internal splinting” OR Achillon OR “small incision surgery” OR “limited access surgery”) AND (“mini-invasive” OR “minimally-invasive” OR “minimum invasive” OR percutaneous OR Griffith OR Griffiths OR Bannister OR Webb OR “keyhole surgery” OR “image-guided surgery”)

**Scopus/ Cochrane Library/ Web of Science/ ClinicalTrials.gov:**

(Achilles OR AATR OR “heel cord” OR “calcaneus tendon”) AND (“mini-open” OR “limited-open” OR MOA OR “minimal incision” OR “mini-incision” OR “internal splinting” OR Achillon OR “small incision surgery” OR “limited access surgery”) AND (“mini-invasive” OR “minimally-invasive” OR “minimum invasive” OR percutaneous OR Griffith OR Griffiths OR Bannister OR Webb OR “keyhole surgery” OR “image-guided surgery”)

**Supplementary Figure 2A.** Funnel plot of re-ruptures showed no definitive evidence of publication bias.


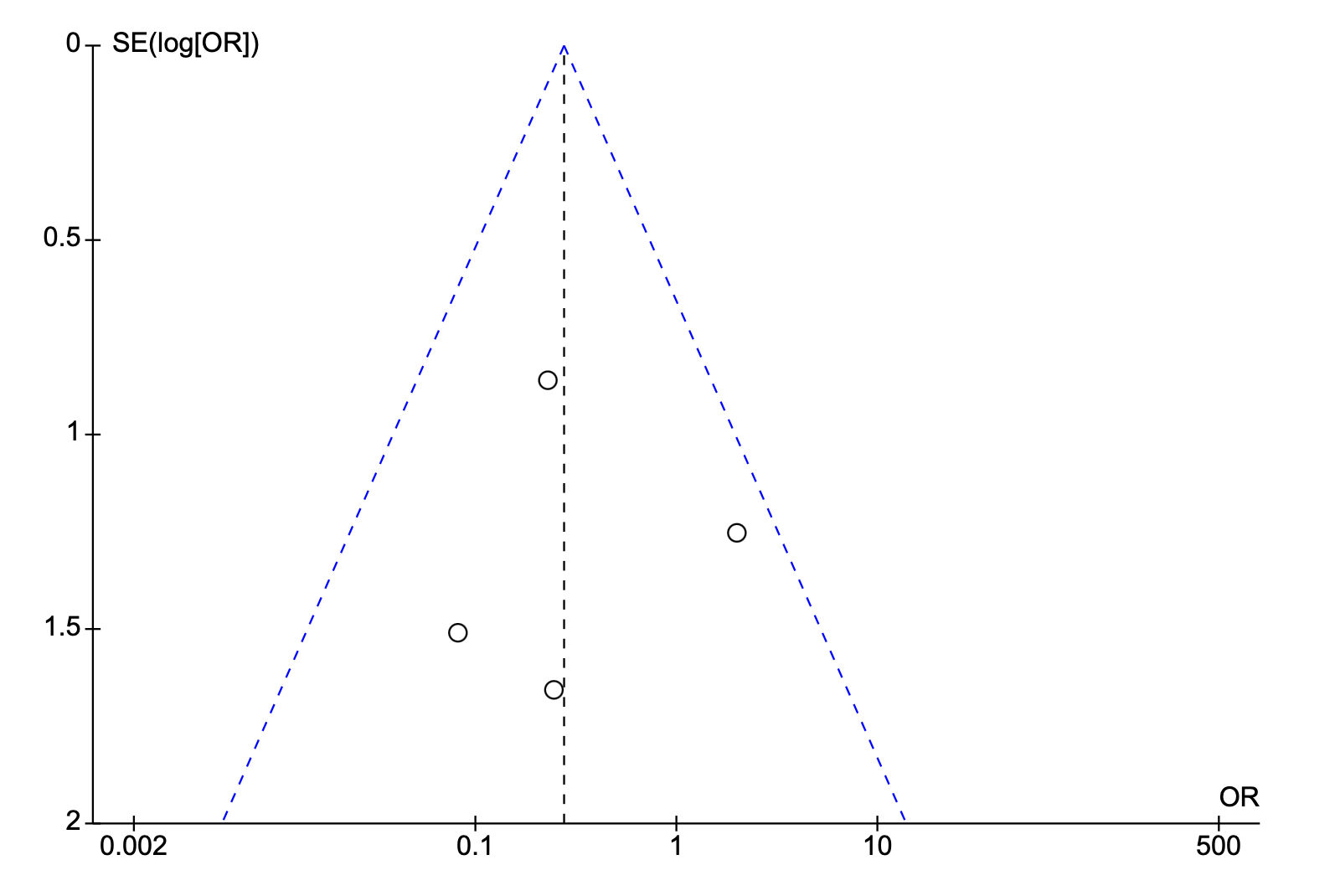


**Supplementary Figure 2B.** Funnel plot of sural nerve injury showed no definitive evidence of publication bias.


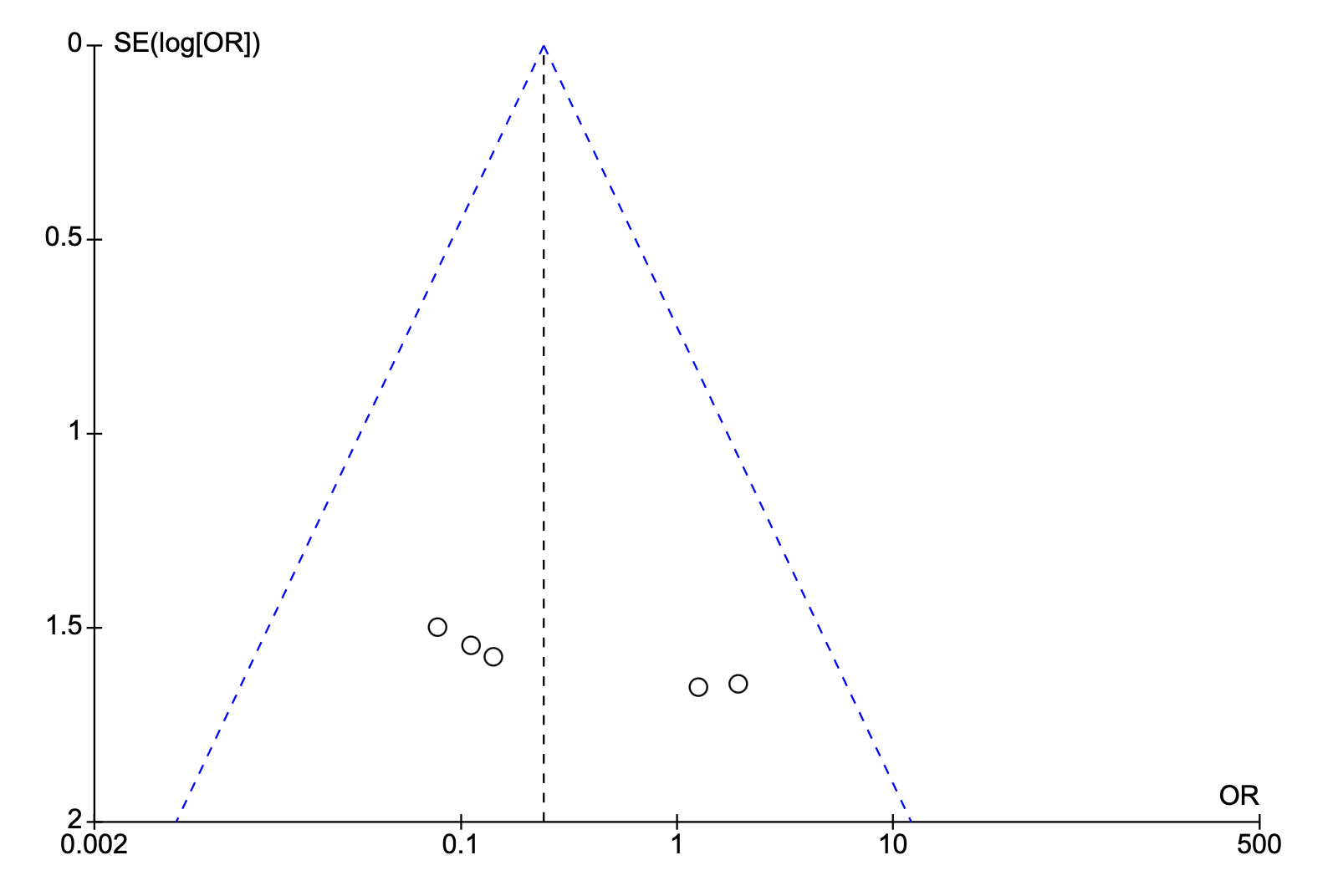


**Supplementary Figure 2C.** Funnel plot of AOFAS Ankle-Hindfoot score showed no definitive evidence of publication bias.


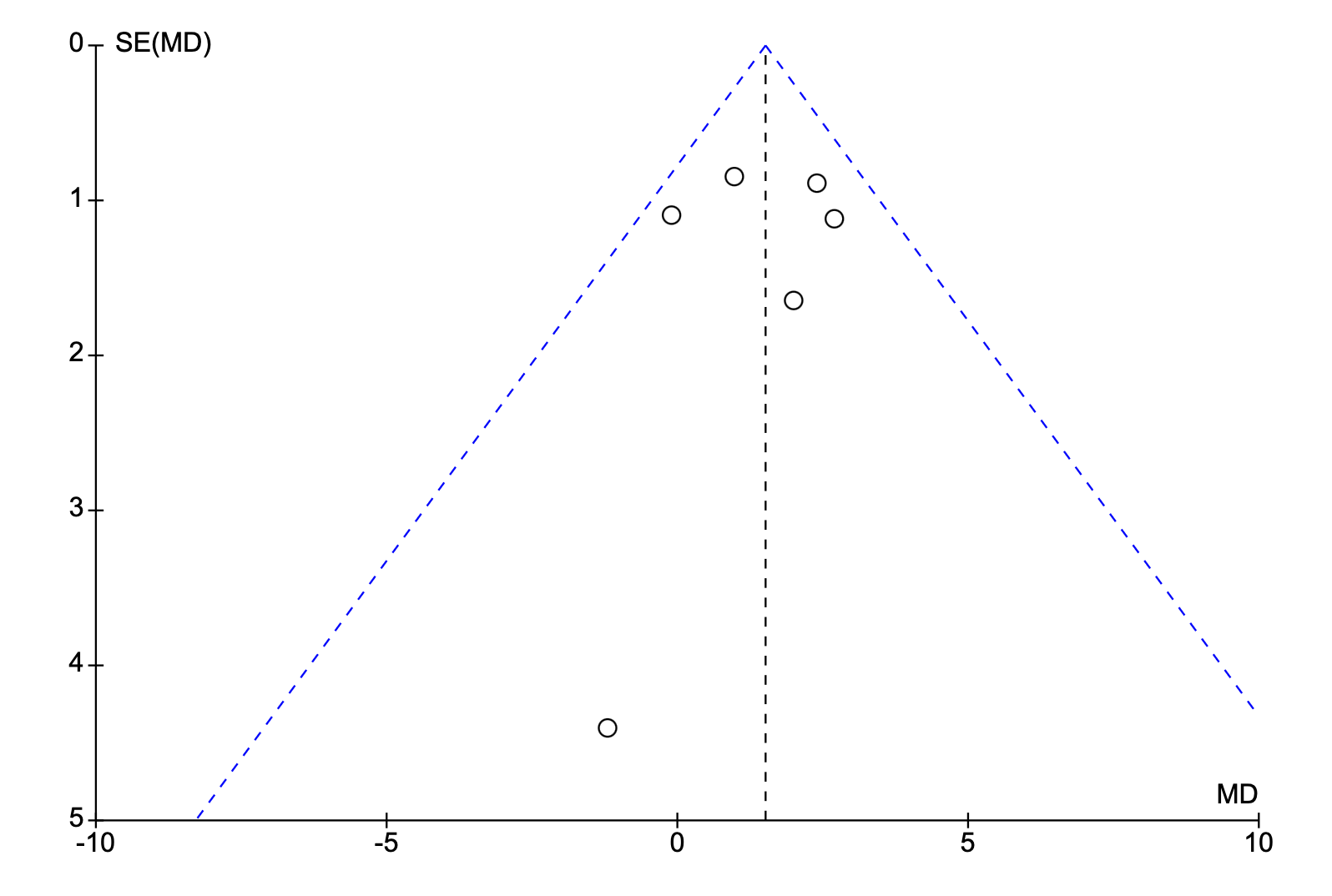

Supplement: Supplementary file 1 — Supplementary Material 1 [file 264_2024_6362_MOESM1_ESM.docx]
